# Effects of *Helicobacter pylori* eradication on the profiles of blood metabolites and their associations with the progression of gastric lesions: a prospective follow-up study

**DOI:** 10.20892/j.issn.2095-3941.2022.0255

**Published:** 2022-08-30

**Authors:** Wenhui Wu, Zongchao Liu, Zhexuan Li, Weidong Liu, Lanfu Zhang, Yang Zhang, Tong Zhou, Weicheng You, Kaifeng Pan, Wenqing Li

**Affiliations:** 1 Key Laboratory of Carcinogenesis and Translational Research (Ministry of Education/Beijing), Department of Cancer Epidemiology, Peking University Cancer Hospital and Institute, Haidian District, Beijing 100142, China; 2 Linqu County Public Health Bureau, Linqu 262600, China; 3 Linqu County People’s Hospital, Linqu 262600, China

**Keywords:** *Helicobacter pylori* infection, gastric cancer, gastric lesion, metabolomics, eradication

## Abstract

**Objective:**

This study aimed at examining the alterations in metabolomic profiles caused by treatment of *H. pylori* infection, and the associations between key plasma metabolites and the risk of gastric lesion progression during follow-up after treatment.

**Methods:**

An intervention trial was performed in 183 participants, 117 of whom were *H. pylori* positive participants receiving treatment for *H. pylori* infection. *H. pylori* positive participants were prospectively followed for 182 to 1,289 days. Untargeted metabolomics assays were conducted on plasma samples collected at baseline, 6 months after treatment, and during continued follow-up.

**Results:**

We identified 59 metabolites with differential posttreatment changes between participants with successful and failed *H. pylori* eradication, 17 metabolites significantly distinguished participants with successful *vs.* failed eradication. Two metabolites [PC(18:1(11Z)/14:1(9Z)) and (2S)-6-amino-2-formamidohexanamide] showed posttreatment changes positively associated with successful *H. pylori* eradication, and were inversely associated with the risk of gastric lesion progression among participants with successful eradication. In contrast, 9-decenoic acid showed posttreatment changes inversely associated with successful eradication: its level was positively associated with the risk of gastric lesion progression among participants with successful eradication. Although the identified metabolites showed a temporary but significant decline after treatment, the trend generally reversed during continued follow-up, and pretreatment levels were restored.

**Conclusions:**

Treatment of *H. pylori* infection significantly altered plasma metabolic profiles in the short term, and key metabolites were capable of distinguishing participants with successful *vs.* failed eradication, but might not substantially affect metabolic regulation in the long term. Several plasma metabolites were differentially associated with the risk of gastric lesion progression among participants with successful or failed eradication.

## Introduction

*Helicobacter pylori* (*H. pylori*), a confirmed oncogenic factor, plays an important role in the development of gastric cancer (GC)^1–3^. Previous studies from our team and others have reported significant decreases in GC incidence and mortality after *H. pylori* eradication^4–6^, thus supporting treatment of *H. pylori* infections as a potential strategy for preventing GC. However, before a major public health campaign can be launched to treat *H. pylori* infections for GC prevention, the full range of beneficial and adverse effects of treatment of *H. pylori* infection must be clarified, and the systemic molecular profiles posttreatment must be elucidated.

Host metabolism regulates whole-body physiology and controls diverse biological functions. Cellular metabolic pathways are extensively involved in the immune system’s defense against pathogens and maintenance of tissue homeostasis^7^. Disruption of the interplay between immune and metabolic responses underlies the emergence of major chronic non-communicable diseases^8^. Notably, metabolic reprogramming is well recognized as an important hallmark of cancer^9–11^. Studies have suggested that perturbations in cellular metabolism are closely associated with the regulation of the gut microbiota by *H. pylori* infection^12,13^. *H. pylori* may sense and respond to host metabolites in the gastric epithelium, thus altering the host metabolic environment and promoting GC development^10,14,15^. Given these interactions between *H. pylori* and the human metabolome, and the roles of metabolite profiles as central regulators of the balance between diseased and healthy states, elucidation of the influence of *H. pylori* eradication on metabolite profiles is essential for understanding the full range of effects. Indeed, previous studies have found that *H. pylori* eradication therapy alters energy metabolism and lipid metabolic homeostasis^16–18^. In addition, the host metabolic environment may influence the efficacy of antibiotic treatment, thus implying that specific metabolites might be beneficial in treating bacterial infections^19^. Clarifying how metabolite profiles may alter the risk of gastric lesion progression after *H. pylori* eradication is also of interest. In-depth investigation of metabolite profiles may enable prediction of the effects of treatment of *H. pylori* infection and long-term gastric lesion outcomes; thus, such research is warranted to facilitate precision GC prevention and control in the future.

To fill these knowledge gaps, we comprehensively examined the alterations in the plasma metabolomic landscape after the treatment of *H. pylori* infection, and evaluated the influences of plasma metabolites on the risk of gastric lesion progression during follow-up. We performed an *H. pylori* intervention trial in Linqu county, Shandong province, China, a well-known high-risk area for GC^4,20^.

## Materials and methods

### Study participants

**Figure 1:**
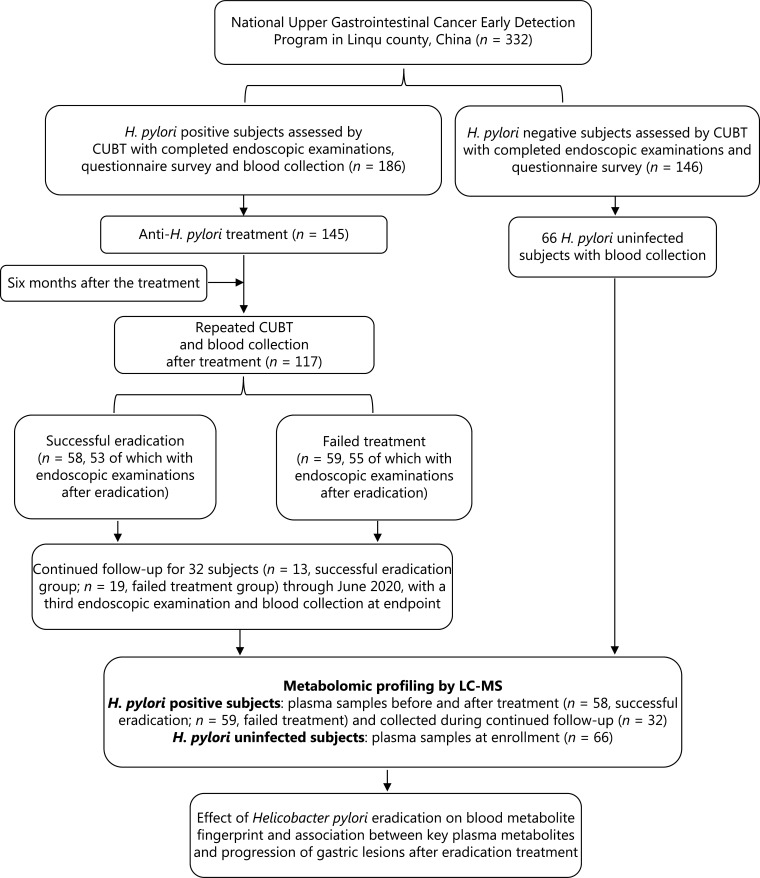
Study design and general workflow. CUBT, ^13^C-urea breath test; *H. pylori*, *Helicobacter pylori*; LC-MS/MS, liquid chromatography-mass spectrometry/mass spectrometry.

The general workflow of this study is shown in **Figure 1**. The study was approved by the Institutional Review Board of Peking University Cancer Hospital. An *H. pylori* intervention trial was conducted within the framework of the National Upper Gastrointestinal Cancer Early Detection Program in Linqu, Shandong province, China. A total of 332 participants, including 186 with *H. pylori* infection and 146 without *H. pylori* infection, as assessed with a ^13^C-urea breath test (CUBT), were enrolled in December 2016. These individuals were asked to consent to endoscopic examinations and a questionnaire survey. A total of 66 *H. pylori* uninfected participants had their blood drawn. Among the 186 *H. pylori* infected participants, 145 agreed to receive a 10-day quadruple anti-treatment for *H. pylori* infection, and 41 were not included in further study because they rejected the treatment. At 6 months after the treatment, CUBTs were conducted again to determine whether the eradication was successful in 117 participants; the results identified 58 participants with successful *H. pylori* eradication and 59 with failed treatment. Among them, 108 participants (53 with successful eradication and 55 with failed eradication) also received endoscopic examination after treatment. These participants were prospectively followed up for 182 to 1,289 days (June 14, 2017 to June 24, 2020), and 32 participants underwent a third endoscopic examination at the end of follow-up, thus resulting in 3 gastric histopathology measurements (at baseline, 6 months after eradication treatment, and at the study endpoint). These 32 participants were prospectively followed up for a median of 534 days [interquartile range (IQR) 490 to 868 days]. A total of 13 participants with successful eradication were followed up for a median of 533 days (IQR 502 to 857 days), and 19 with failed treatment were followed up for a median of 534 days (IQR 490 to 892 days). The *H. pylori* uninfected individuals were not followed up with repeated endoscopic examinations.

During endoscopic examinations, biopsies were collected at 5 standardized sites and other sites with suspicious lesions, if present. Each participant was assigned a global diagnosis of superficial gastritis, chronic atrophic gastritis, intestinal metaplasia, low-grade intraepithelial neoplasia, high-grade intraepithelial neoplasia, or invasive GC, defined as the most severe gastric histology among all biopsies, according to the criteria of the Updated Sydney System and the Chinese Association of Gastric Cancer^21^. We did not include any participants with normal gastric mucosa, because few of the Linqu residents had completely normal histology^4,21^. Participants were considered to have progression of gastric lesions if the severity of gastric lesions was higher than that in the preceding endoscopic examination. Details regarding gastroscopic examinations have been described in a previous study^22^.

### Sample preparation for metabolomic profiling

Each participant had a 5 mL blood sample collected through a standardized process^23^. A 50 µL plasma sample was collected and transferred to a microcentrifuge tube for preparation. After addition of 200 µL of extract solution (acetonitrile:methanol = 1:1, containing isotopically labeled internal standard mixture), the samples were vortexed for 30 s, sonicated for 10 min in an ice-water bath, and incubated for 1 h at −40°C to precipitate proteins. The samples were centrifuged at 12,000 rpm for 15 min at 4°C, and the resulting supernatants were transferred to a fresh glass vial for analysis.

### Untargeted metabolomic profiling

Liquid chromatography-mass spectrometry/mass spectrometry (LC-MS/MS) analyses were conducted for untargeted metabolomic profiling with an UHPLC system (Vanquish, Thermo Fisher Scientific) with a UPLC BEH Amide column (2.1 mm × 100 mm, 1.7 µm) coupled to a Q Exactive HFX mass spectrometer (Orbitrap MS, Thermo). The mobile phase consisted of 25 mmol/L ammonium acetate and 25 ammonia hydroxide in water (pH = 9.75) (A) and acetonitrile (B). The analysis was performed with an elution gradient as follows: 0–0.5 min, 95% B; 0.5–7.0 min, 95%–65% B; 7.0–8.0 min, 65%–40% B; 8.0–9.0 min, 40% B; 9.0–9.1 min, 40%–95% B; and 9.1–12.0 min, 95% B. The flow rate was 0.5 mL/min. The column temperature was 30°C. The auto-sampler temperature was 4°C, and the injection volume was 2 µL.

The QE HFX mass spectrometer was used because of its ability to acquire MS/MS spectra in information-dependent acquisition mode through the acquisition software (Xcalibur, Thermo Fisher Scientific). In this mode, the acquisition software continuously evaluated the full MS spectrum scan. The ESI source conditions were set as follows: sheath gas flow rate, 30 Arb; auxiliary gas flow rate, 25 Arb; capillary temperature, 350°C; full MS resolution, 60,000; MS/MS resolution, 7,500; collision energy, 10/30/60 in NCE mode; and spray voltage, 3.6 kV (positive) or −3.2 kV (negative). The raw data were derived in mzXML format with ProteoWizard and were processed with in-house software for peak detection, extraction, alignment, and integration. An in-house MS2 database was applied for metabolite annotation. The cutoff value for annotation was set at 0.3.

Quality control (QC) samples were prepared by mixture of equal aliquots of the supernatants from all samples. Strict criteria were followed to ensure high data quality.

### Bioinformatics and statistical analysis

For all metabolites, the plasma levels at baseline (pretreatment), posttreatment (6-month), and continued follow-up endpoints were log-transformed and standardized before analyses. We also calculated the posttreatment fold change (PFC) for each metabolite, defined as the ratio of the 6-month posttreatment to pretreatment levels.

### Identification of key metabolites with significant PFCs associated with successful *H. pylori* eradication

**Figure 2:**
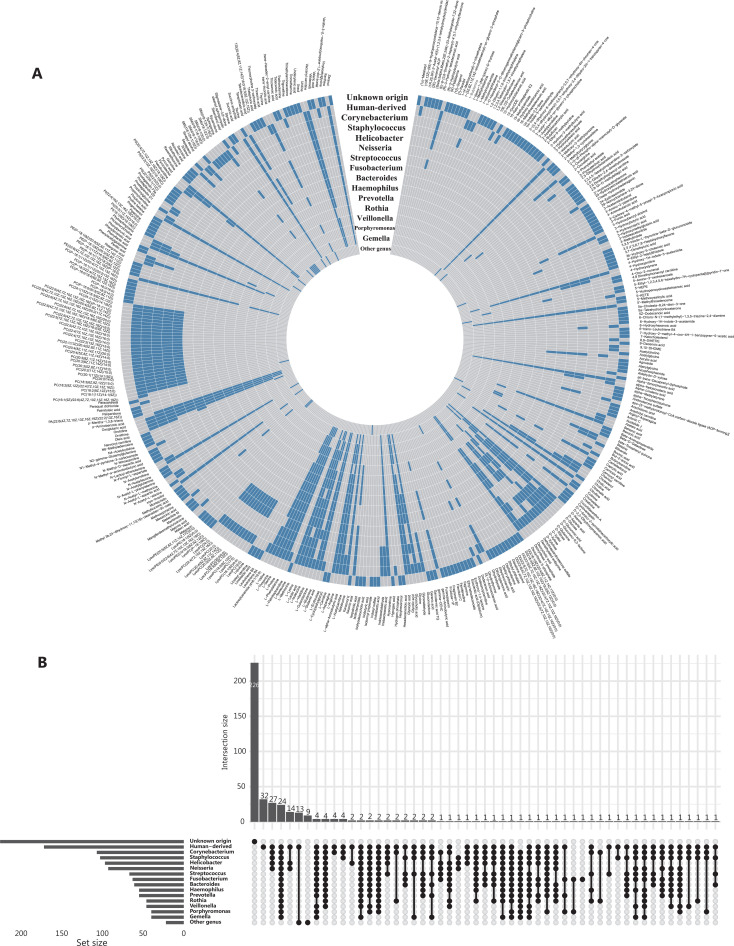
Plasma metabolites of human-origin and (or) gut bacterial origin. (A) Circular heatmap of metabolites (*n* = 413) in 16 categories. Each layer of the ring represents a category, and each vertical row represents a metabolite (*n* = 413). Metabolites belonging to a specific category are indicated by blue boxes on the layer corresponding to that category. (B) Upset plot showing overlap of metabolite origin.

Given significant effects of the intestinal microbiota on blood metabolites and host physiology^24,25^, we focused on metabolites of bacterial origin and categorized them into those associated with *Helicobacter* and those associated with other genera but not *Helicobacter*, on the basis of the prior knowledge of gut flora metabolism from the Kyoto Encyclopedia of Genes and Genomes (KEGG) database and literature research (**Figure 2A, 2B**). We then used the sparse group least absolution shrinkage and selection operator (LASSO) regression model to identify metabolites with differing PFCs between the groups with successful and failed *H. pylori* eradication. Sparse group LASSO is a method of regression analysis that determines important predictors in terms of both feature groups and individual features^26^. Orthogonal partial least squares-discriminant analysis (OPLS-DA) was applied to the identified metabolites, to visualize and distinguish patients with successful eradication *vs.* failed treatment. We further assessed the effect magnitudes by calculating odds ratios (ORs) and their 95% confidence intervals (CIs) for the associations of these metabolites with treatment success (successful eradication *vs.* treatment failure), with logistic regression models adjusting for age and gender. On the basis of linear discriminant analyses (LDA), the metabolites significantly associated with treatment outcome were combined to assess individuals’ potential for successful eradication. Beyond the PFCs of metabolite levels, we examined whether the baseline metabolite levels might be associated with the treatment outcome.

### Evaluation of relationships between metabolites associated with successful eradication and the risk of gastric lesion progression

We assessed whether the identified metabolites associated with successful eradication in the above analyses correlated with the risk of gastric lesion progression during follow-up. Logistic regression models adjusting for age, gender, and baseline pathology were used for the association analyses of baseline metabolite levels (*n* = 108). We also used the multiple time-point endoscopic examinations and metabolite measurements for 32 participants to examine the associations between changes in metabolite levels and the evolution of gastric lesions during follow-up, by using the generalized estimating equations (GEE) model^27^ adjusting for age, gender, and baseline pathology. The “exchangeable” correlation structure and identity link function were applied in the GEE model. We compared the metabolite levels over time for *H. pylori* positive participants, as well as *H. pylori* uninfected participants, with the Wilcoxon rank sum test.

Two-sided *P* < 0.05 was considered to indicate a statistically significant association.

## Results

### Study characteristics and QC measurements

Our study included a total of 183 participants (**Supplementary Table S1**). All QC samples were distributed within 2 standard deviations of the first component in the principal component analysis (**Supplementary Figure S1A and S1B**) and were closely aggregated on the 2-dimensional principal component analysis coordinate (**Supplementary Figure S1C and S1D**), thus suggesting high data quality. The pairwise Spearman correlations between QC samples ranged from 0.88 to 0.98 (**Supplementary Figure S1E and S1F**), thus indicating the high stability of the analytical process. The median relative standard deviation of peak areas of internal standards was 6.9% and 7.6% for the positive and negative mode, respectively, thus indicating high reproducibility. A total of 413 metabolites were identified, of which 155 and 172 were considered bacterially derived and human-derived metabolites (140 overlapping in 2 categories), respectively. Most bacterially derived metabolites were associated with 2 or more genera. For example, 95 of 97 *Helicobacter*-derived metabolites were also found to be consistent with other genus origins (**Figure 2A**).

### Changes in metabolite levels after successful *H. pylori* eradication

**Figure 3:**
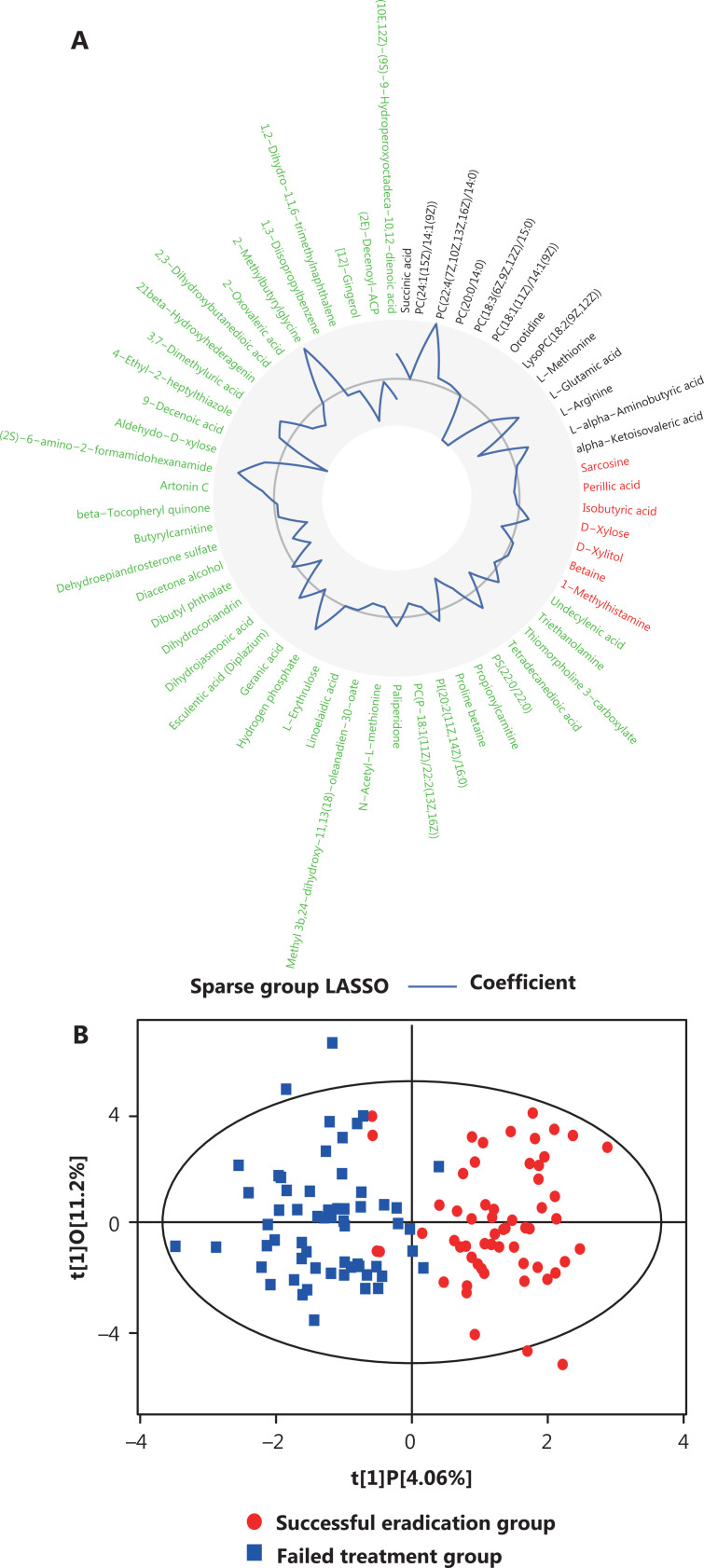
Plasma metabolites with differing posttreatment changes distinguishing groups with successful eradication *vs.* failed treatment. (A) Circular line-plot of 59 metabolites identified by the sparse group LASSO model. The sparse group LASSO model identified 59 plasma metabolites with differential posttreatment changes in levels distinguishing cases of successful from failed eradication. The circular line-plot represents regression coefficients of the key metabolites. The inner circle in gray is a reference line for the coefficient equal to 0. The blue polyline indicates the magnitude of coefficients in the positive (away from the center) or negative (toward the center) direction. The metabolites that are *Helicobacter*-associated (black label), non-*Helicobacter*-associated (red label), and of other/unknown origin (green label) are colored differently. (B) Score plot of OPLS-DA indicating adequate performance of the 59 key metabolites in distinguishing groups with successful eradication *vs.* failed treatment. In OPLS-DA, 117 participants are projected to 2-dimensional coordinates, on the basis of the 59 metabolites’ posttreatment changes in metabolic levels. Participants with successful eradication are indicated by red circles, and failed cases are indicated by blue squares. LASSO, least absolution shrinkage and selection operator regression; OPLS-DA, orthogonal partial least squares-discriminant analysis.

On the basis of the sparse group LASSO model, we identified 59 metabolites with differential PFCs in the plasma between participants with successful eradication and failed treatment. Of these, 20 had known bacterial origin (13 *Helicobacter*-derived and 7 non-*Helicobacter*-derived metabolites) (**Figure 3A**). On the basis of these 59 metabolites, OPLS-DA analyses indicated distinct posttreatment changes in metabolic profiles between participants with successful eradication *vs.* failed treatment (**Figure 3B**).

**Figure 4:**
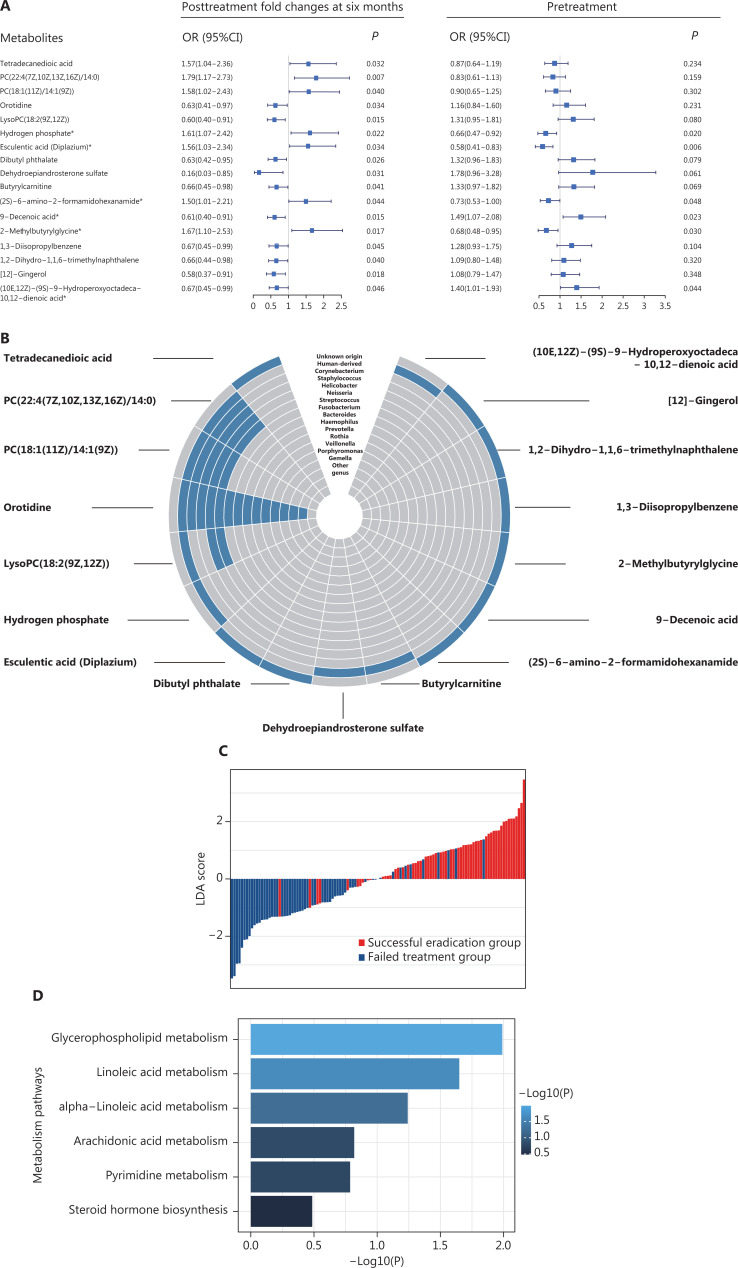
Plasma metabolites significantly associated with successful *H. pylori* eradication. (A) The 17 metabolites significantly associated with treatment outcome (successful eradication *vs.* treatment failure). ORs (95% CIs) and *P* values for the associations between successful eradication and posttreatment changes (left) and baseline levels (right) of metabolites were calculated with logistic regression models adjusting for age and gender. Metabolites with significant associations for both posttreatment changes and pretreatment levels are marked with asterisks. (B) Circular heatmap of 17 metabolites with gut bacterial origin. (C) LDA scores integrating the posttreatment changes in 17 metabolites, indicating individuals’ potential for successful eradication. LDA models were used on the basis of the posttreatment changes in 17 metabolites that were significantly associated with the treatment outcome. The LDA scores are shown in red for participants with successful eradication and in blue for participants with failed treatment. (D) KEGG pathway analysis for 17 metabolites. *P* values for enriched pathways are shown in horizontal bars. CI, confidence interval; KEGG, Kyoto Encyclopedia of Genes and Genomes; LDA, linear discriminant analyses; OR, odds ratio.

Among the 59 metabolites, the PFCs of 17 metabolites were significantly associated with the odds of successful eradication (*P* < 0.05), on the basis of logistic regression analyses. Of those, the PFCs of 7 metabolites were positively associated with successful *H. pylori* eradication (OR > 1), and 10 were inversely associated (OR < 1) (**Figure 4A**). Four of the 17 metabolites—PC(22:4(7Z,10Z,13Z,16Z)/14:0), PC(18:1(11Z)/14:1(9Z)), orotidine, and lysoPC(18:2(9Z,12Z))—were known to be of bacterial origin and to be *Helicobacter* associated (**Figure 4B**). Individuals’ potential for successful eradication was visualized with the LDA model. Integration of the PFCs for these 17 metabolites distinguished participants with successful eradication from those with failed treatment (**Figure 4C**). We also tested the associations in baseline (pretreatment) levels, which indicated statistically significant associations for 6 metabolites (*P* < 0.05). Among them, we observed inverse associations between successful eradication and the PFCs of 2 metabolites [9-decenoic acid and (10E,12Z)-(9S)-9-hydroperoxyoctadeca-10,12-dienoic acid], and positive associations with their baseline (pretreatment) levels. In contrast, the PFCs of 4 metabolites—hydrogen phosphate, esculentic acid (diplazium), (2S)-6-amino-2-formamidohexanamide {also called apo-[3-methylcrotonoyl-CoA:carbon-dioxide ligase (ADP-forming)]}, and 2-methylbutyrylglycine—were positively associated with successful eradication, and their baseline levels were inversely associated with successful eradication (**Figure 4A**).

Pathway analysis indicated that glycerophospholipid metabolism (*P* = 0.010) and linoleic acid metabolism (*P* = 0.022) were among the top pathways significantly associated with successful eradication (**Figure 4D**).

### Association between metabolites and the risk of gastric lesion progression among participants with successful eradication and failed treatment

We further examined the associations between the 17 metabolites with statistically significant PFCs after successful eradication (**Figure 4A**) and the risk of gastric lesion progression during follow-up. Logistic regression models adjusting for age, gender, and pretreatment histopathology indicated that PC(18:1(11Z)/14:1(9Z)) and (2S)-6-amino-2-formamidohexanamide were inversely associated with the risk of gastric lesion progression (OR < 1), and 9-decenoic acid was positively associated with gastric lesion progression (OR > 1), but only among participants with successful eradication (**Supplementary Table S2** and **Table 1**). In addition, we used GEE models leveraging the multi-time point metabolite measurement and endoscopic follow-up data, which longitudinally corroborated the associations between these metabolites and gastric lesion progression (β coefficient < 0 and > 0 indicating inverse and positive associations, respectively) (**Supplementary Table S2** and **Table 1**), thus suggesting potential statistical interactions of these metabolites and treatment of *H. pylori* infection in the modulation of the risk of gastric lesion progression (**Table 1**).

**Table 1:** Plasma metabolites differentially associated with gastric lesion progression in participants with successful eradication and failed treatment^a^

Metabolites	Logistic regression				*P* for interaction	Generalized estimating equations				*P* for interaction
	Successful eradication group (*n* = 53)		Failed treatment group (*n* = 55)			Successful eradication group (*n* = 13)		Failed treatment group (*n* = 19)		
	OR (CI)	*P*	OR (CI)	*P*		Estimate (CI)	*P*	Estimate (CI)	*P*	
PC(18:1(11Z)/14:1(9Z))	0.58 (0.34, 0.99)	0.048	0.83 (0.40, 1.72)	0.337	0.063	−0.660 (−1.290, −0.031)	0.042	0.102 (−0.358, 0.563)	0.357	0.055
(2S)-6-amino-2-formamidohexanamide	0.55 (0.30, 1.00)	0.050	2.56 (1.15, 5.73)	0.027	0.009	−0.627 (−1.217, 0.038)	0.040	0.132 (−0.206, 0.470)	0.261	0.024
9-Decenoic acid	2.13 (1.01, 4.47)	0.048	0.86 (0.44, 1.68)	0.357	0.032	0.705 (0.120, 1.289)	0.024	−0.049 (−0.475, 0.377)	0.425	0.055

^a^Among 17 individual metabolites with significant posttreatment changes identified by sparse group LASSO and logistic regression models, 3 metabolites had differential association with gastric lesion progression in participants with successful eradication *vs*. failed treatment. Analysis was performed with logistic regression and generalized estimating equations adjusting for age, gender, and baseline pathology. Logistic regression analyses were conducted to examine the progression of gastric lesions when gastric lesions at the baseline and endpoint were considered. Generalized estimating equation analyses were conducted to examine the progression of gastric lesions according to the trajectory of gastric lesions during follow-up.

### Comparisons of selected metabolite levels at baseline, after treatment, and during continued follow-up

**Figure 5:**
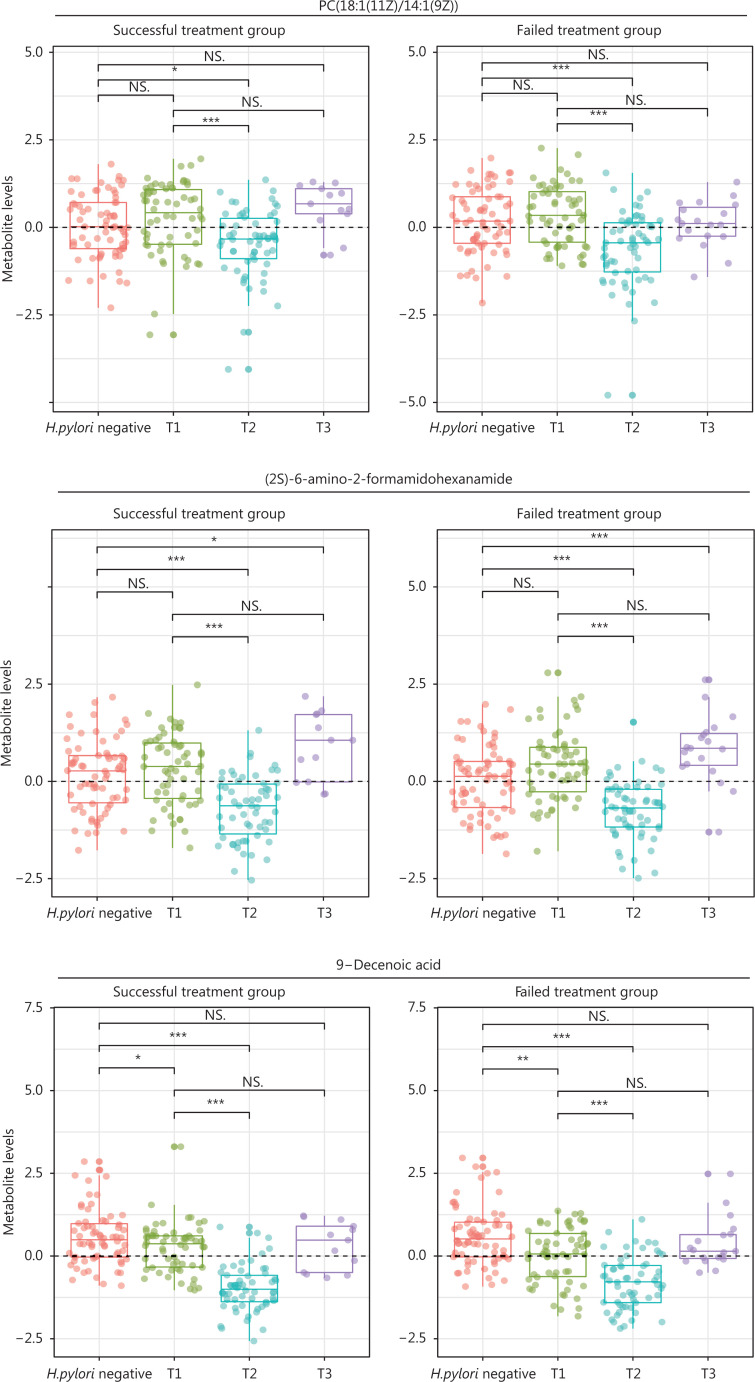
Levels of selected metabolites at baseline (pretreatment), posttreatment, and the endpoint during follow-up among *H. pylori* infected participants, and at baseline for *H. pylori* uninfected participants. Three metabolites that had posttreatment changes significantly associated with successful eradication and were associated with the risk of gastric lesion progression are shown. For *H. pylori* infected participants, metabolite levels at 3 time-points—pretreatment (T1), posttreatment (6 months after treatment, T2), and the follow-up endpoint (T3)—are shown for participants with successful eradication (*n* = 13) and failed treatment (*n* = 19). The group of *H. pylori* uninfected participants (*n* = 66) serves as a reference for comparison with the Wilcoxon rank sum test. Wilcoxon rank sum tests were also conducted for the comparisons of metabolite levels at posttreatment (T2) and the follow-up endpoint (T3) with the baseline (T1). NS., not significant; *, *P* < 0.05; **, *P* < 0.01; ***, *P* < 0.001; *H. pylori*, *Helicobacter pylori*.

For 17 metabolites whose PFCs at 6 months were significantly associated with successful eradication, we plotted the levels at baseline (T1, pretreatment), 6-month posttreatment (T2), and the endpoint during follow-up (T3) for *H. pylori* infected participants, and those at baseline for *H. pylori* uninfected participants. The 3 aforementioned metabolites showed significantly decreased levels after treatment of *H. pylori* infection (T2 *vs.* T1), and the decreasing trend was reversed during continued follow-up (T3 *vs.* T1). The changing patterns for each specific metabolite appeared similar between successful eradication (left panels, **Figure 5**) and failed treatment groups (right panels, **Figure 5**), although different PFC magnitudes were observed between groups. However, each group also displayed heterogeneous changes in metabolite levels that represented 3 different patterns. *H. pylori* uninfected participants showed similar baseline levels of PC(18:1(11Z)/14:1(9Z)) and (2S)-6-amino-2-formamidohexanamide to those in infected participants, but had significantly higher 9-decenoic acid than infected participants. The declining posttreatment trends for 3 metabolites (T2 *vs.* T1; T2 *vs. H. pylori* negative) were all reversed during continued follow-up (T3 *vs.* T1), but (2S)-6-amino-2-formamidohexanamide rose to a level significantly higher than that in *H. pylori* negative participants (T3 *vs. H. pylori* negative). PC(18:1(11Z)/14:1(9Z)) and 9-decenoic acid increased, such that the levels at the endpoint were similar to the baseline levels (T3 *vs.* T1) (**Figure 5**). The other 14 metabolites generally followed the patterns of the 3 identified metabolites, although butyrylcarnitine showed a continued decreasing trend during follow-up among participants with failed treatment (**Supplementary Figure S2**).

## Discussion

In this study, we identified a panel of metabolites with differential PFCs between participants with successful *H. pylori* eradication and participants with failed treatment. Of these, 17 metabolites significantly distinguished participants with successful eradication from those with failed treatment. PC(18:1(11Z)/14:1(9Z)) and (2S)-6-amino-2-formamidohexanamide had PFCs positively associated with successful *H. pylori* eradication and were inversely associated with the risk of gastric lesion progression among participants with successful eradication. In contrast, the PFC of 9-decenoic acid was inversely associated with successful eradication and was positively associated with gastric lesion progression among participants with successful eradication. The levels of the identified metabolites temporarily significantly declined after treatment but then rose during continued follow-up and returned to at least pretreatment levels.

The effects of *H. pylori* eradication on GC prevention have been recognized. Our long-term follow-up study in the Shandong Intervention Trial reported decreased GC incidence and mortality^4^. However, little is known regarding the full range of beneficial and adverse effects of treatment of *H. pylori* infection. We previously reported the restoration of gastric microbiota by *H. pylori* eradication to a status similar to that in uninfected individuals, thus indicating beneficial effects on the gut microbiota^28^. The effect of treatment of *H. pylori* infection on host metabolism also must be clarified, because the metabolic system is a key regulator of biological functions.

Prior studies based on limited metabolite coverage have reported altered metabolism after *H. pylori* eradication. For example, Fang *et al.*^16^ have reported significantly decreased trimethylamine N-oxide and creatine in *H. pylori*-infected children after *H. pylori* eradication. Orihara *et al*.^18^ have found changes in gastric mucosal phosphatidylcholine and its fatty acid composition after *H. pylori* eradication (*n* = 8 successful eradication). The only available study on untargeted fecal lipidomics and plasma metabolomics has revealed potential effects of *H. pylori* eradication on host energy and lipid metabolism; however, that study was based on a limited sample size (*n* = 29) and did not involve any participants with failed treatment^17^. Therefore, major knowledge gaps regarding the effects of treatment of *H. pylori* infection on global metabolomic profiles remain to be filled. Metabolomic reprogramming is a key hallmark of carcinogenesis^9–11^, and metabolic alterations have been associated with the risk of GC in previous studies^22,29^. However, whether metabolite signatures may predict the risk of gastric lesion progression also remains to be clarified. Connecting *H. pylori* eradication-associated metabolic alterations and the risk of progression of gastric lesions to GC has major public health implications in informing targeted primary prevention of GC in the future.

In our study, the sparse group LASSO model was initially applied, and we identified 59 metabolites with differential PFCs between the successful eradication and failed treatment groups. We leveraged knowledge of metabolites of bacterial and human origin for categorization, given the significant changes in the microbiota caused by *H. pylori* eradication^28^. Further analyses based on multivariate-adjusted logistic regression models corroborated the findings for 17 key metabolites whose PFCs were significantly associated with successful eradication. Pathways involving glycerophospholipid and linoleic acid metabolism were mainly enriched, thus revealing that lipid metabolism might be altered after successful eradication. However, whether the effect was caused by the medications used for eradication or the changes in *H. pylori* infection status is unknown.

We identified 3 successful eradication-associated metabolites that were further associated with the risk of gastric lesion progression among participants with successful eradication. Among them, PC(18:1(11Z)/14:1(9Z)) can be derived from humans, *Helicobacter*, *Staphylococcus*, *Corynebacterium*, and *Neisseria*; is involved in the glycerophospholipid metabolism pathway; and plays a role in promoting intracellular cholesterol trafficking and membrane lipid homeostasis^30^. In contrast to previously reported inverse associations between *H. pylori* infection and gastric mucosal PCs^31,32^, our analysis of baseline data did not support associations between *H. pylori* infection and PC(18:1(11Z)/14:1(9Z)). Nonetheless, treatment of *H. pylori* infection led to a significant decrease in this metabolite, thus possibly suggesting alternative effects caused by the eradication of other genera during treatment or the effects of medication on the human body.

Both (2S)-6-amino-2-formamidohexanamide and 9-decenoic acid are exogenous metabolites. Whereas 9-decenoic acid has been identified to be present in yeasts^33^, the bacterial origin of (2S)-6-amino-2-formamidohexanamide remains unknown. The carboximidic acid (2S)-6-amino-2-formamidohexanamide participates in biotin metabolism. Previous studies have shown that biotin deficiency enhances inflammation and certain chronic inflammatory diseases^34^. The medium-chain fatty acid 9-decenoic acid is a primary contributor to the β-oxidation of fatty acids^35–37^, an oxidation process that is elevated in many cancers^35,36^. We found significant associations between *H. pylori* infection and 9-decenoic acid, but not with (2S)-6-amino-2-formamidohexanamide. Levels of both metabolites significantly decreased after treatment of *H. pylori* infection, but whether the posttreatment effects on these 2 metabolites were due to the medication itself or to other possible reasons warrants further investigation.

To our knowledge, the present study is among the first to demonstrate potential interactions between the treatment of *H. pylori* infection and metabolites in influencing the risk of gastric outcomes. Previous studies have reported a wide spectrum of PCs that are significantly lower in patients with GC^38,39^, but evidence for the other 2 metabolites was sparse. Further studies are needed to clarify the relationships of (2S)-6-amino-2-formamidohexanamide and 9-decenoic acid with the gastric microbiota. We found that the risk of gastric lesion progression was significantly inversely associated with PC(18:1(11Z)/14:1(9Z)) and (2S)-6-amino-2-formamidohexanamide, and positively associated with 9-decenoic acid, but only among participants with successful treatment. These results may suggest that plasma metabolites modify the long-term effects of *H. pylori* eradication, and also provide evidence of the potential roles of the 3 metabolites in forecasting the gastric outcomes of *H. pylori* eradication.

The identified metabolites all declined after treatment of *H. pylori* infection, although the magnitude of the decrease varied in participants with successful eradication and failed treatment. The PFCs of PC(18:1(11Z)/14:1(9Z)) and (2S)-6-amino-2-formamidohexanamide were positively associated with successful eradication: higher PFCs were observed among participants with successful eradication than those with failed treatment. In contrast, the posttreatment change in 9-decenoic acid was inversely associated with successful eradication, thus indicating greater PFCs among participants with failed treatment. Whether the effect was caused by the eradication of other genera or the effects of medication on the human body remains to be clarified. However, during continued follow-up, the decreasing trend in metabolite levels was generally reversed, and the levels returned to at least pretreatment values. Therefore, our data demonstrated that the changes in metabolomic profiles caused by *H. pylori* eradication, if any, would tend to be temporary and might not lead to long-lasting metabolic deregulation in the long term.

Our study was based on an intervention trial conducted in a recognized high-risk area for GC with a relatively large sample size. Prospective endoscopic follow-up and multi-time point sample collection after treatment of *H. pylori* infection are notable features of this study, which enabled the assessment of alterations in global metabolomic profiles caused by *H. pylori* eradication and of whether metabolite signatures alter the risk of gastric lesion progression during prolonged follow-up. We acknowledge several study limitations. First, we conducted only 1 trial and were not able to validate the findings. Ideally, targeted metabolomic profiling with absolute quantification of metabolites would have been performed in an independent cohort. Second, we did not examine the altered metabolomic profiles in gastric tissues and other biological specimens. Third, the *H. pylori* negative participants were not prospectively followed up, and only the baseline plasma samples were subjected to LC-MS/MS assays. Fourth, the study may not directly indicate the biological mechanisms underlying the interplay among *H. pylori* eradication and metabolite signatures, and its effect on the progression of gastric lesions.

## Conclusions

In conclusion, plasma metabolic profiles were significantly altered after *H. pylori* eradication, and key metabolites were capable of distinguishing participants with successful eradication from those with failed treatment. Several plasma metabolites were differentially associated with the risk of gastric lesion progression among participants with successful *vs.* failed eradication. The posttreatment changes in metabolites generally reversed during continued follow-up, thus suggesting that *H. pylori* eradication may not substantially affect metabolic regulation in the long term. Our study provides novel insights into the metabolomic alterations caused by treatment of *H. pylori* infection, thereby adding to knowledge regarding the full range of effects of treatment of *H. pylori* infection. Further large-scale studies are needed to validate our findings.
