# Supplementary material for: Effects of Helicobacter pylori eradication on the profiles of blood metabolites and their associations with the progression of gastric lesions: a prospective follow-up study
Source: Cancer Biol Med. 2022 Aug 30;19(8):1259–73. doi: 10.20892/j.issn.2095-3941.2022.0255 (PMC9425181; doi:10.20892/j.issn.2095-3941.2022.0255)
Supplement: Supplementary file 1 [file cbm-19-1259-s001.pdf]

Supplementary materials

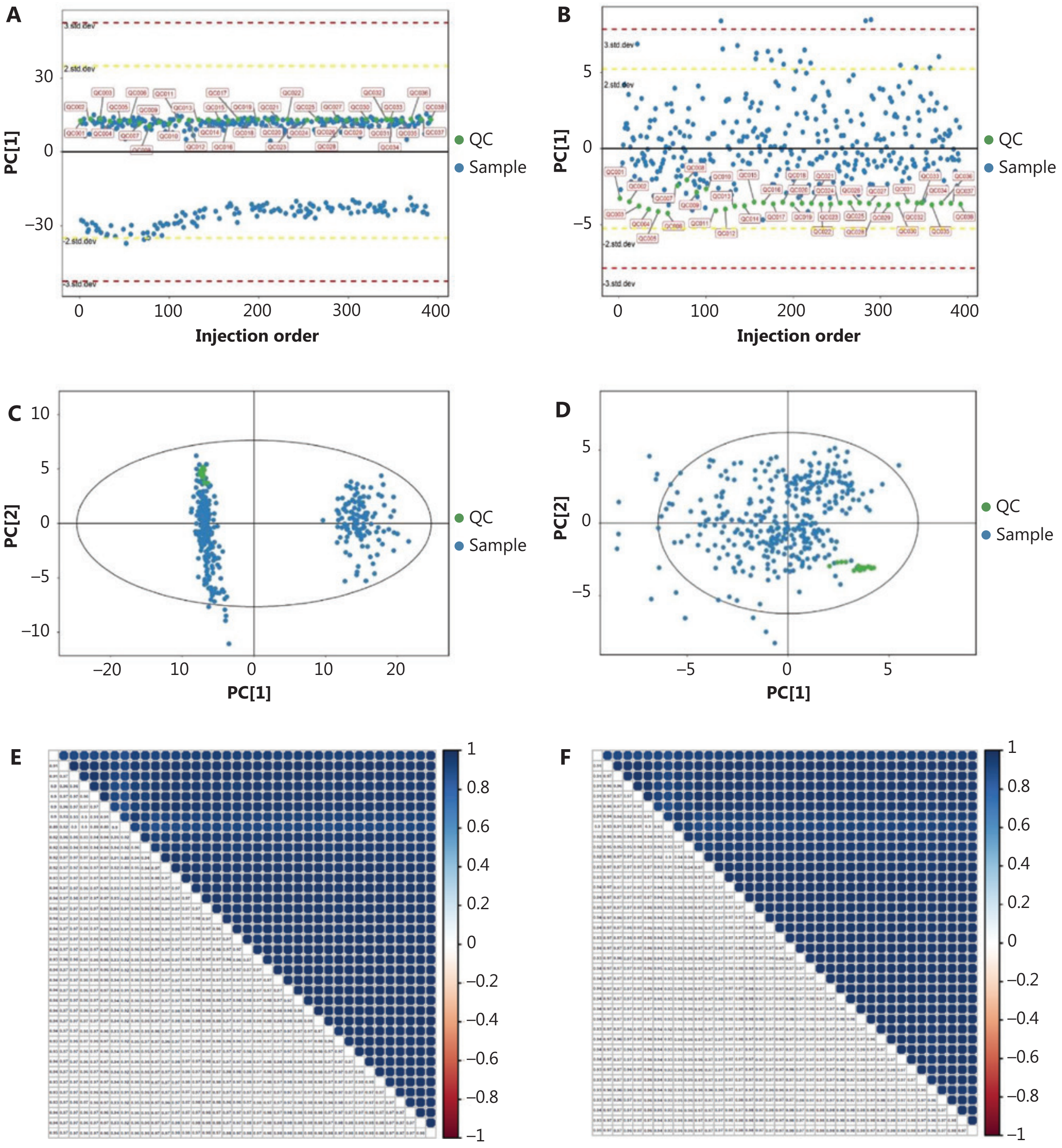

Figure S1 QC for the LC-MS/MS analysis. (A) Distribution of the first component in PCA for QC samples in positive electrospray ionization mode. (B) Distribution of the first component in PCA for QC samples in negative electrospray ionization mode. In panels (A) and (B), the horizontal axis refers to the injection order and the vertical axis refers to the first principle component in PCA. The standard deviation of the first component is indicated by dash lines. The green dots represent the QC samples and the blue dots represent the tested samples. (C) PCA score plot for QC samples in positive electrospray ionization mode. (D) PCA score plot for QC samples in negative electrospray ionization mode. In panel (C) and (D), the horizontal and vertical axes refer to the first and second principle components in PCA, respectively. The green dots represent the QC samples and the blue dots represent the tested samples. (E) Pairwise Spearman's correlation analysis for QC samples in

positive electrospray ionization mode. (F) Pairwise Spearman's correlation analysis for QC samples in negative electrospray ionization mode. Spearman's correlation coefficients were calculated for pairwise correlation of QC samples. The magnitude of correlation coefficients varies by color, with the blue and red colors indicating positive and negative correlation, respectively.

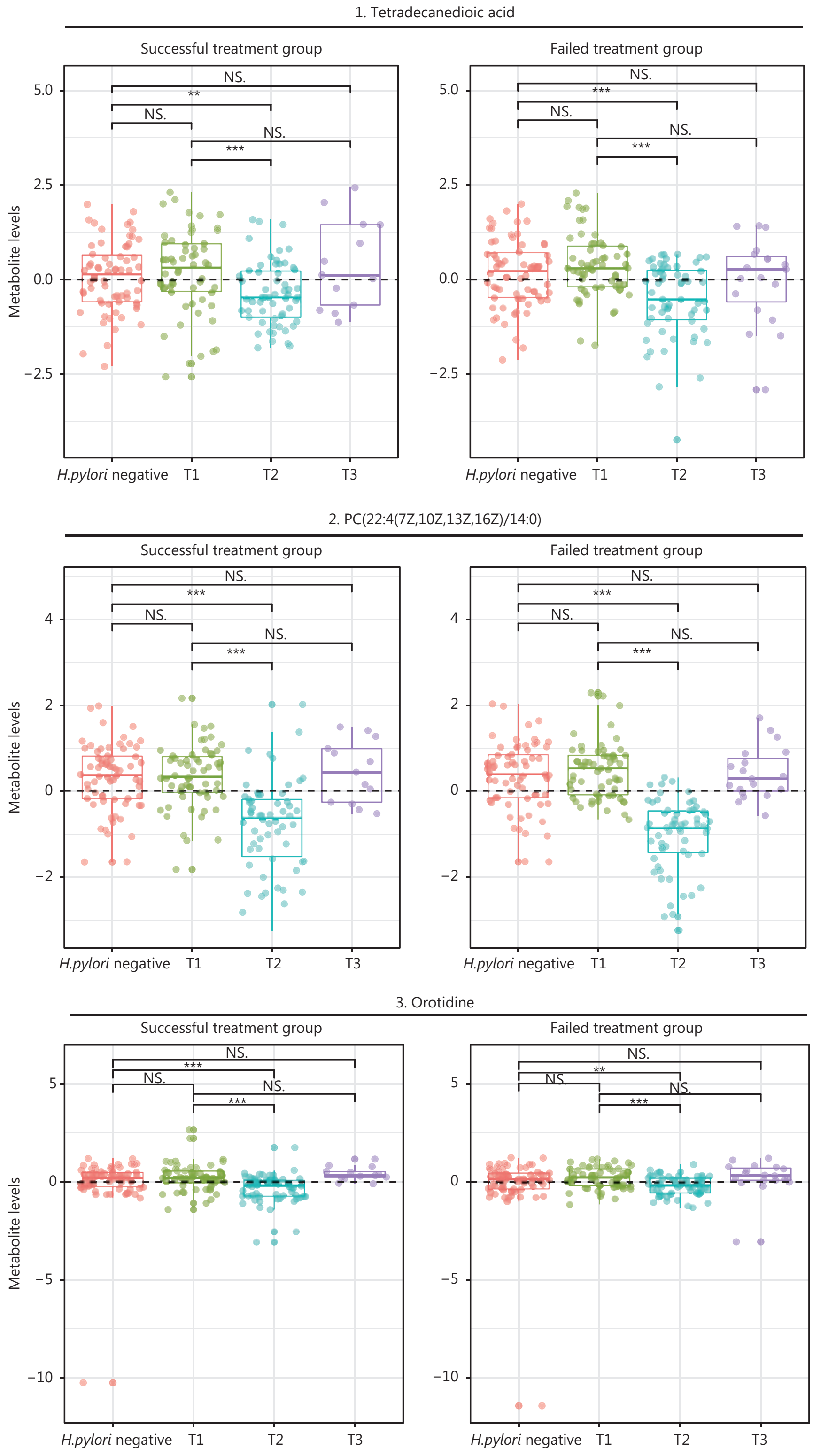

Figure S2 Continued.

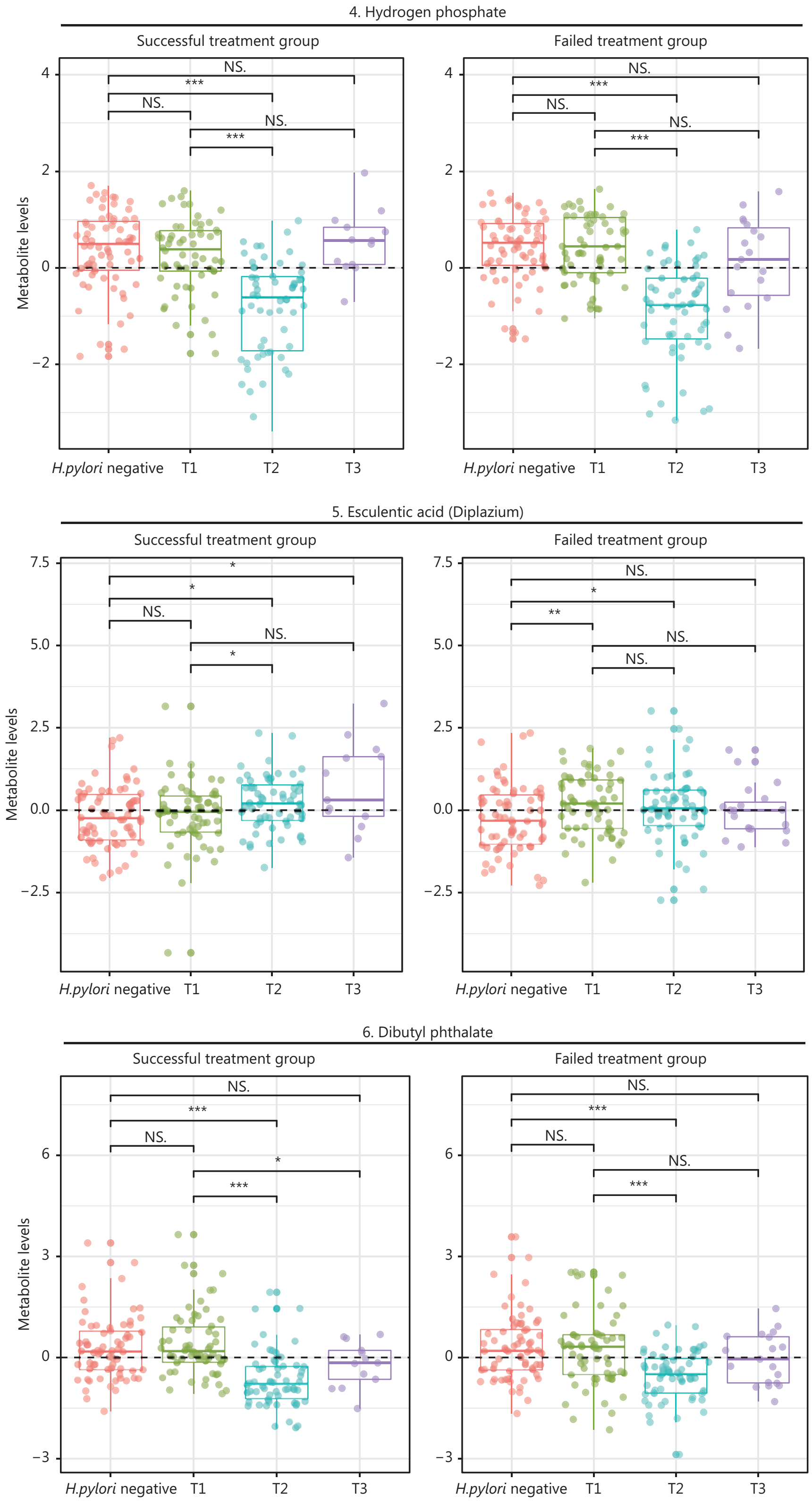

Figure S2 Continued.

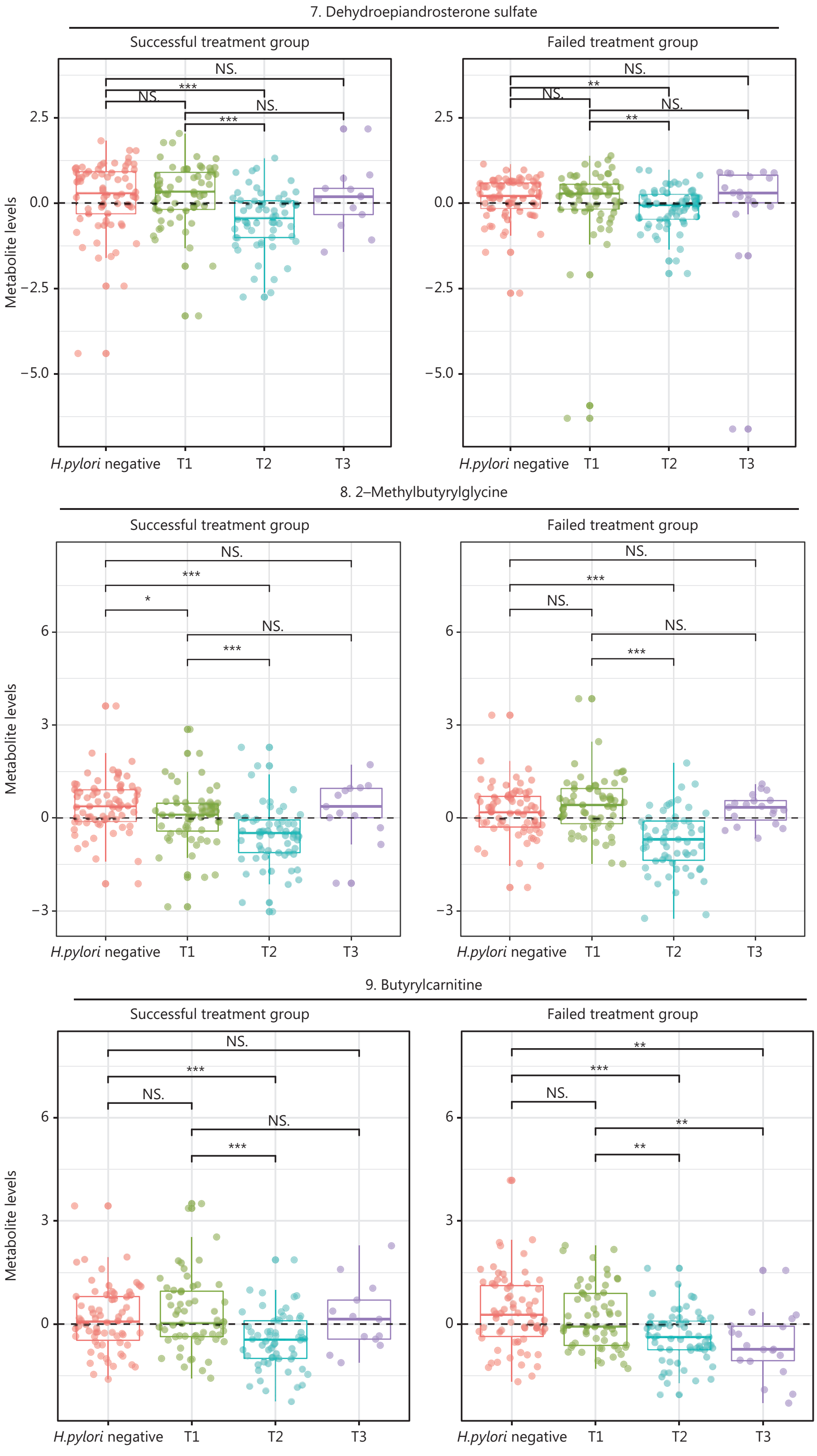

Figure S2 Continued.

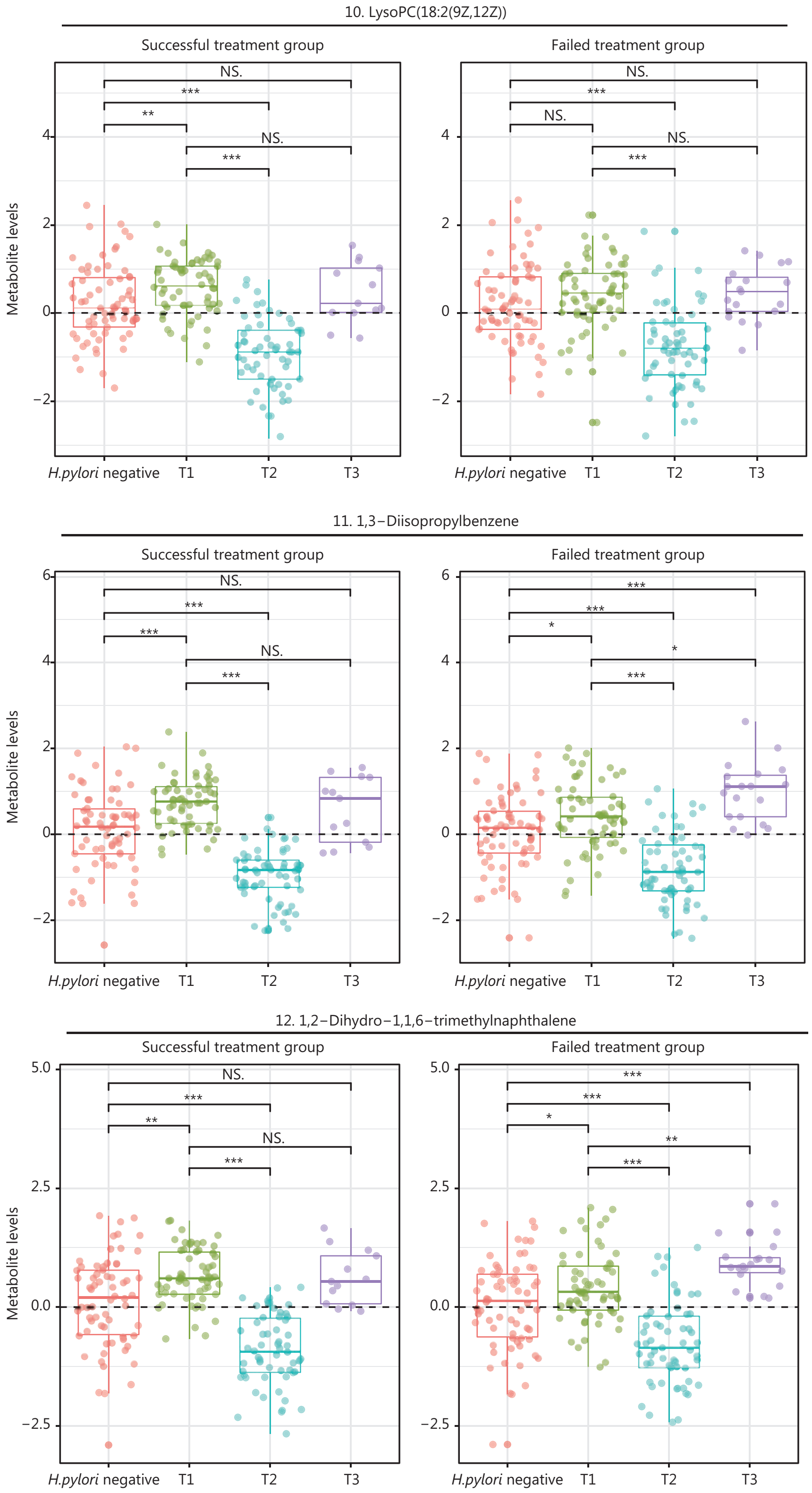

Figure S2 Continued.

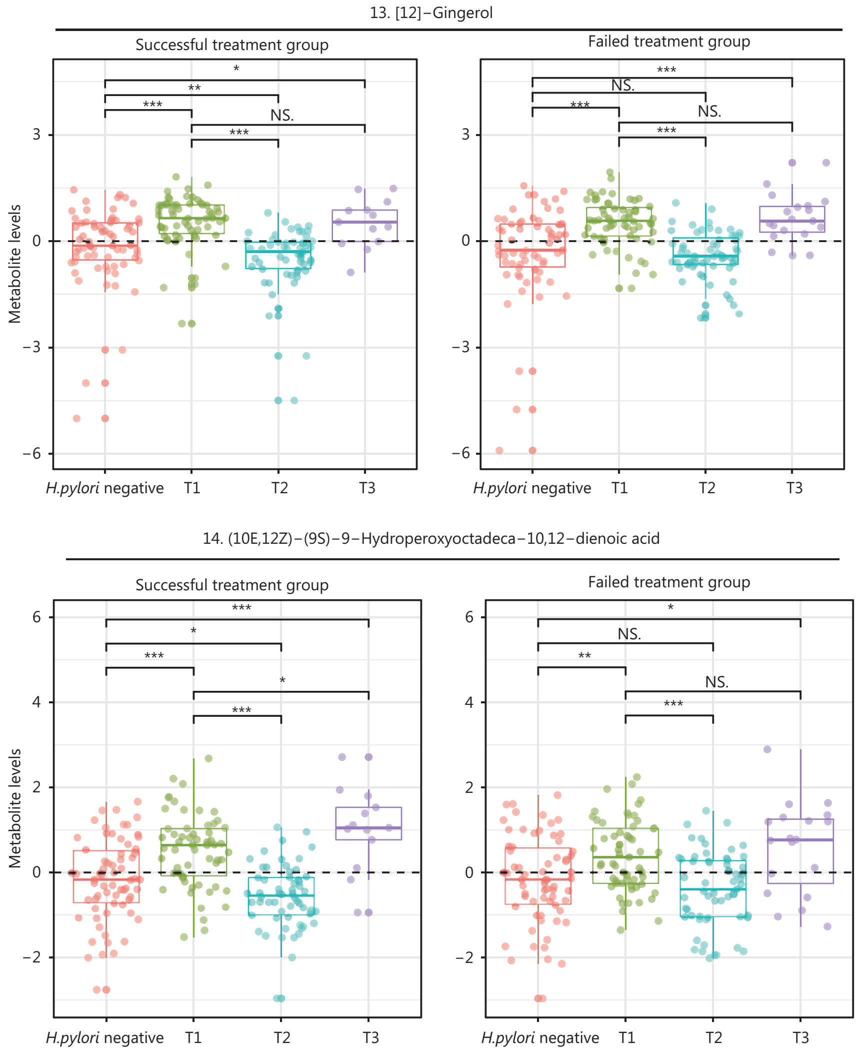

Figure S2 Levels of 14 other selected metabolites at baseline (pretreatment), posttreatment, and endpoint during follow-up for *H. pylori* infected participants, and at baseline for *H. pylori* uninfected participants. Except the three metabolites shown in **Figure 5**, other 14 metabolites which had posttreatment changes significantly associated with successful eradication are shown. For *H. pylori* infected participants, metabolite levels at 3 time-points—pretreatment (T1), posttreatment (6 months after treatment, T2), and the follow-up endpoint (T3)—are shown for participants with successful eradication (n = 13) and failed treatment (n = 19). The group of *H. pylori* uninfected participants (n = 66) serves as a reference for comparison with the Wilcoxon rank sum test. Wilcoxon rank sum tests were also conducted for the comparisons of metabolite levels at posttreatment (T2) and the follow-up endpoint (T3) with the baseline (T1). The changing metabolite levels basically followed three different patterns represented by the three highlighted metabolites in our manuscript (**Figure 5**). Metabolite 1~9 basically had the pattern similar with PC(18:1(11Z)/14:1(9Z), but butyrylcarnitine (metabolite 9) showed continued decreasing level during follow-up for the failed treatment group. Metabolite 10 had the pattern similar with 9-decenoic acid. Metabolite 11 to 14 had the pattern similar with (2S)-6-amino-2-formamidohexanamide. NS., not significant; *P < 0.05; **P < 0.01; ***P < 0.001.

Table S1 Baseline characteristics of study participants

Characteristics	*H. pylori* positive participants							*H. pylori* negative participants		
	Successful eradication (*n* = 58)			Failed treatment (*n* = 59)			P^b	*n* = 66	P^c	P^d
	Total (*n* = 58)	With continued follow-up (*n* = 13)[a]	Without continued follow-up (*n* = 45)	Total (*n* = 59)	With continued follow-up (*n* = 19)[a]	Without continued follow-up (*n* = 40)				
Age (mean ± SD)[e]	56.5 ± 9.2	60.4 ± 6.6	55.4 ± 9.5	56.3 ± 8.9	56.6 ± 8.5	56.2 ± 9.1	0.899	56.9 ± 7.5	0.829	0.712
Gender (%)[f]							0.011		0.486	0.055
Male	28 (48.3%)	7 (53.8%)	21 (46.7%)	42 (71.2%)	15 (78.9%)	27 (67.5%)		36 (54.5%)		
Female	30 (51.7%)	6 (46.2%)	24 (53.3%)	17 (28.8%)	4 (21.1%)	13 (32.5%)		30 (45.5%)		
BMI, kg/m^2 (mean ± SD)[e]	26.3 ± 1.2	26.1 ± 1.0	26.4 ± 1.3	26.2 ± 1.3	26.4 ± 1.2	26.2 ± 1.4	0.835	24.7 ± 2.5	< 0.001	< 0.001
Smoking (%)[f]							0.883		0.142	0.104
No	42 (72.4%)	9 (69.2%)	33 (73.3%)	42 (71.2%)	12 (63.2%)	30 (75.0%)		55 (83.3%)		
Yes	16 (27.6%)	4 (30.8%)	12 (26.7%)	17 (28.8%)	7 (36.8%)	10 (25.0%)		11 (16.7%)		
Alcohol consumption (%)[f]							0.06		0.859	0.076
No	43 (74.1%)	10 (76.9%)	33 (73.3%)	34 (57.6%)	8 (42.1%)	26 (65.0%)		48 (72.7%)		
Yes	15 (25.9%)	3 (23.1%)	12 (26.7%)	25 (42.4%)	11 (57.9%)	14 (35.0%)		18 (27.3%)		
Antibiotic use (%)[f]							0.401		0.391	0.104
No	47 (81.0%)	10 (76.9%)	37 (82.2%)	44 (74.6%)	13 (68.4%)	31 (77.5%)		41 (62.1%)		
Yes	11 (19.0%)	3 (23.1%)	8 (17.8%)	15 (25.4%)	6 (31.6%)	9 (22.5%)		6 (9.1%)		
Missing	0 (0.0%)	0 (0.0%)	0 (0.0%)	0 (0.0%)		0 (0.0%)		19 (28.8%)		
Gastric lesions (%)[g]							0.247		0.138	0.001
SG	15 (25.9%)	1 (7.7%)	14 (31.1%)	7 (11.9%)	0 (0.0%)	7 (17.5%)		21 (31.8%)		
CAG	12 (20.7%)	1 (7.7%)	11 (24.4%)	12 (20.3%)	6 (31.6%)	6 (15.0%)		19 (28.8%)		
IM	25 (43.1%)	8 (61.5%)	17 (37.8%)	31 (52.5%)	9 (47.4%)	22 (55.0%)		25 (37.9%)		
DYS	6 (10.3%)	3 (23.1%)	3 (6.7%)	9 (15.3%)	4 (21.1%)	5 (12.5%)		1 (1.5%)		

[a]Participants with continued follow-up had endoscopic examinations at pretreatment (T1), posttreatment 6 months after treatment (T2), and the follow-up endpoint (T3).
[b]Successful eradication group *vs.* failed treatment group.
[c]Successful eradication group *vs.* *H. pylori* negative group.
[d]Failed treatment group *vs.* *H. pylori* negative group.
[e]*t*-test.
[f]$\chi 2$ test.
[g]Fisher's exact test.
CAG, chronic atrophic gastritis; *H. pylori*, *Helicobacter pylori*; IM, intestinal metaplasia; DYS, dysplasia; SG, superficial gastritis.

Table S2 Associations of pretreatment levels of the 59 key plasma metabolites with the risk of gastric lesion progression

Metabolites	Logistic regression				Generalized estimating equations			
	Successful eradication group (n = 53)		Failed treatment group (n = 55)		Successful eradication group (n = 13)		Failed treatment group (n = 19)	
	OR (95% CI)	*P*	OR (95% CI)	*P*	β coefficient (95% CI)	*P*	β coefficient (95% CI)	*P*
Undecylenic acid	1.50 (0.87, 2.58)	0.108	0.70 (0.34, 1.43)	0.205	0.444 (0.018, 0.869)	0.043	−0.634 (−1.019, −0.249)	0.003
Triethanolamine	0.51 (0.26, 0.98)	0.046	1.13 (0.57, 2.21)	0.386	−0.418 (−1.013, 0.178)	0.124	0.213 (−0.251, 0.677)	0.225
Thiomorpholine 3-carboxylate	0.50 (0.26, 0.97)	0.042	3.14 (1.20, 8.20)	0.025	0.442 (0.012, 0.872)	0.045	−0.478 (−0.989, 0.033)	0.062
Tetradecanedioic acid	1.53 (0.88, 2.65)	0.101	0.64 (0.30, 1.35)	0.161	0.320 (−0.481, 1.122)	0.256	0.116 (−0.321, 0.552)	0.332
Succinic acid	0.73 (0.40, 1.34)	0.199	0.57 (0.24, 1.34)	0.140	−0.041 (−0.798, 0.715)	0.464	−0.200 (−0.667, 0.267)	0.241
Sarcosine	0.35 (0.19, 0.66)	0.003	0.81 (0.44, 1.49)	0.283	−0.717 (−1.272, −0.162)	0.017	−0.274 (−0.773, 0.224)	0.183
PS(22:0/22:0)	0.82 (0.45, 1.51)	0.299	2.50 (1.15, 5.41)	0.026	−0.03 (−0.525, 0.465)	0.461	−0.038 (−0.473, 0.397)	0.443
Propionylcarnitine	0.84 (0.50, 1.43)	0.300	0.68 (0.34, 1.35)	0.176	0.137 (−0.686, 0.961)	0.392	0.024 (−0.430, 0.478)	0.465
Proline betaine	0.80 (0.46, 1.37)	0.245	1.19 (0.67, 2.11)	0.309	−0.088 (−0.525, 0.350)	0.371	−0.224 (−0.641, 0.193)	0.188
PI(20:2(11Z,14Z)/16:0)	0.69 (0.39, 1.22)	0.140	1.08 (0.57, 2.04)	0.424	0.412 (−0.104, 0.928)	0.094	0.020 (−0.412, 0.452)	0.470
Perillic acid	1.05 (0.60, 1.84)	0.444	0.71 (0.35, 1.45)	0.215	0.338 (−0.139, 0.815)	0.122	−0.324 (−0.742, 0.095)	0.102
PC(P-18:1(11Z)/22:2(13Z,16Z))	1.01 (0.62, 1.66)	0.482	1.63 (0.64, 4.16)	0.194	0.544 (0.088, 1.000)	0.025	−0.711 (−1.210, −0.211)	0.010
PC(24:1(15Z)/14:1(9Z))	1.19 (0.75, 1.89)	0.263	1.33 (0.60, 2.96)	0.278	−0.750 (−1.443, −0.056)	0.038	−0.154 (−0.615, 0.308)	0.292
PC(22:4(7Z,10Z,13Z,16Z)/14:0)	0.80 (0.48, 1.32)	0.231	0.71 (0.32, 1.56)	0.237	−0.250 (−0.582, 0.082)	0.108	0.252 (−0.239, 0.743)	0.199
PC(20:0/14:0)	0.38 (0.14, 1.02)	0.053	0.74 (0.23, 2.39)	0.338	0.116 (−0.187, 0.419)	0.265	−0.653 (−1.192, −0.114)	0.023
PC(18:3(6Z,9Z,12Z)/15:0)	1.28 (0.70, 2.34)	0.252	1.10 (0.45, 2.71)	0.431	0.232 (−0.164, 0.628)	0.168	0.210 (−0.236, 0.657)	0.219
PC(18:1(11Z)/14:1(9Z))	0.58 (0.34, 0.99)	0.048	0.83 (0.40, 1.72)	0.337	−0.660 (−1.290, −0.031)	0.042	0.102 (−0.358, 0.563)	0.357
Paliperidone	1.04 (0.66, 1.63)	0.449	0.74 (0.33, 1.65)	0.268	0.009 (−0.433, 0.451)	0.486	0.382 (−0.302, 1.065)	0.179
Orotidine	0.90 (0.56, 1.45)	0.362	1.15 (0.48, 2.71)	0.397	−0.116 (−0.564, 0.332)	0.335	−0.492 (−0.866, −0.118)	0.015
N-Acetyl-L-methionine	0.88 (0.50, 1.55)	0.359	0.94 (0.53, 1.67)	0.430	−0.255 (−0.899, 0.390)	0.258	0.195 (−0.181, 0.571)	0.197
Methyl 3b,24-dihydroxy-11,13(18)-oleanadien-30-oate	1.17 (0.68, 2.01)	0.312	0.51 (0.19, 1.40)	0.136	0.023 (−0.516, 0.563)	0.471	−0.164 (−0.607, 0.280)	0.272
LysoPC(18:2(9Z,12Z))	1.08 (0.62, 1.88)	0.412	1.09 (0.63, 1.89)	0.399	0.140 (−0.363, 0.643)	0.324	0.030 (−0.523, 0.583)	0.465
Linoelaidic acid	1.08 (0.61, 1.91)	0.411	0.96 (0.49, 1.88)	0.463	0.554 (0.069, 1.039)	0.030	0.096 (−0.347, 0.539)	0.361
L-Methionine	1.43 (0.75, 2.74)	0.180	1.51 (0.76, 3.03)	0.163	0.356 (−0.451, 1.162)	0.234	0.312 (−0.201, 0.824)	0.159

Table S2 Continued

Metabolites	Logistic regression				Generalized estimating equations			
	Successful eradication group (n = 53)		Failed treatment group (n = 55)		Successful eradication group (n = 13)		Failed treatment group (n = 19)	
	OR (95% CI)	P	OR (95% CI)	P	β coefficient (95% CI)	P	β coefficient (95% CI)	P
L-Glutamic acid	0.74 (0.42, 1.30)	0.190	0.36 (0.12, 1.03)	0.054	0.184 (−0.276, 0.644)	0.255	0.136 (−0.366, 0.638)	0.328
L-Erythrulose	1.60 (0.89, 2.87)	0.093	0.91 (0.50, 1.68)	0.403	−0.054 (−0.570, 0.461)	0.431	−0.339 (−0.750, 0.072)	0.087
L-Arginine	0.88 (0.48, 1.61)	0.364	1.63 (0.86, 3.09)	0.103	0.592 (0.188, 0.996)	0.008	−0.132 (−0.696, 0.431)	0.350
L-alpha-Aminobutyric acid	0.84 (0.50, 1.41)	0.295	5.33 (1.59, 17.83)	0.011	−0.382 (−0.898, 0.134)	0.112	0.187 (−0.353, 0.727)	0.285
Isobutyric acid	1.64 (0.88, 3.07)	0.096	0.91 (0.50, 1.66)	0.399	0.561 (−0.055, 1.177)	0.067	0.059 (−0.392, 0.510)	0.415
Hydrogen phosphate	0.87 (0.49, 1.57)	0.353	0.36 (0.14, 0.95)	0.042	−0.102 (−0.793, 0.589)	0.404	−0.298 (−0.702, 0.106)	0.112
Geranic acid	1.58 (0.82, 3.02)	0.125	1.16 (0.60, 2.24)	0.355	0.484 (−0.050, 1.018)	0.068	−0.398 (−0.880, 0.083)	0.087
Esculentic acid (Diplazium)	0.67 (0.37, 1.22)	0.134	0.83 (0.39, 1.76)	0.343	−0.128 (−0.589, 0.332)	0.323	0.270 (−0.211, 0.750)	0.178
Dihydrojasmonic acid	3.11 (1.24, 7.76)	0.021	1.02 (0.32, 3.27)	0.489	−0.233 (−0.474, 0.007)	0.055	−0.261 (−0.828, 0.307)	0.225
Dihydrocoriandrin	1.34 (0.66, 2.73)	0.251	0.75 (0.37, 1.49)	0.245	0.278 (−0.249, 0.805)	0.193	0.473 (−0.003, 0.948)	0.051
Dibutyl phthalate	1.21 (0.70, 2.09)	0.279	0.73 (0.40, 1.33)	0.196	−0.043 (−0.689, 0.602)	0.456	0.095 (−0.344, 0.534)	0.361
Diacetone alcohol	0.98 (0.65, 1.49)	0.469	0.81 (0.43, 1.53)	0.296	−0.005 (−0.897, 0.886)	0.496	0.018 (−0.366, 0.402)	0.469
Dehydroepiandrosterone sulfate	0.68 (0.21, 2.21)	0.297	2.16 (0.45, 10.3)	0.209	−0.640 (−1.695, 0.415)	0.159	−0.011 (−0.420, 0.398)	0.483
D-Xylose	1.79 (0.90, 3.56)	0.080	0.85 (0.43, 1.65)	0.340	0.172 (−0.318, 0.662)	0.282	−0.289 (−0.791, 0.214)	0.173
D-Xylitol	3.08 (0.61, 15.57)	0.126	0.79 (0.55, 1.13)	0.139	0.643 (0.291, 0.995)	0.001	0.152 (−0.308, 0.612)	0.294
Butyrylcarnitine	1.09 (0.70, 1.71)	0.371	1.41 (0.62, 3.21)	0.249	0.014 (−0.472, 0.500)	0.481	0.259 (−0.313, 0.832)	0.228
Betaine	1.94 (1.06, 3.53)	0.035	1.82 (0.82, 4.02)	0.107	0.131 (−0.362, 0.624)	0.331	−0.066 (−0.542, 0.410)	0.410
beta-Tocopheryl quinone	1.87 (1.06, 3.29)	0.035	3.17 (1.05, 9.60)	0.043	−0.258 (−0.798, 0.283)	0.217	−0.603 (−1.041, −0.165)	0.012
Artonin C	1.18 (0.73, 1.91)	0.289	1.00 (0.54, 1.88)	0.496	0.155 (−0.363, 0.672)	0.311	0.126 (−0.480, 0.732)	0.366
(2S)-6-amino-2-formamidohexanamide	0.55 (0.30, 1.00)	0.050	2.56 (1.15, 5.73)	0.027	−0.627 (−1.217, 0.038)	0.040	0.132 (−0.206, 0.470)	0.261
alpha-Ketoisovaleric acid	0.78 (0.44, 1.41)	0.248	0.60 (0.26, 1.38)	0.155	−0.263 (−0.762, 0.235)	0.192	0.319 (−0.217, 0.856)	0.164
Aldehydo-D-xylose	1.20 (0.64, 2.26)	0.314	1.17 (0.59, 2.31)	0.355	0.143 (−0.380, 0.666)	0.326	−0.282 (−0.731, 0.166)	0.150
9-Decenoic acid	2.13 (1.01, 4.47)	0.048	0.86 (0.44, 1.68)	0.357	0.705 (0.120, 1.289)	0.024	−0.049 (−0.475, 0.377)	0.425

Table S2 Continued

Metabolites	Logistic regression				Generalized estimating equations			
	Successful eradication group (n = 53)		Failed treatment group (n = 55)		Successful eradication group (n = 13)		Failed treatment group (n = 19)	
	OR (95% CI)	*P*	OR (95% CI)	*P*	β coefficient (95% CI)	*P*	β coefficient (95% CI)	*P*
4-Ethyl-2-heptylthiazole	1.63 (0.96, 2.75)	0.063	0.78 (0.38, 1.64)	0.294	0.223 (−0.454, 0.901)	0.294	0.516 (0.135, 0.897)	0.013
3,7-Dimethyluric acid	0.93 (0.54, 1.61)	0.414	0.51 (0.24, 1.09)	0.073	0.292 (−0.178, 0.761)	0.153	−0.034 (−0.494, 0.426)	0.452
21beta-Hydroxyhederagenin	0.91 (0.36, 2.28)	0.433	1.50 (0.66, 3.39)	0.208	−0.497 (−1.185, 0.191)	0.117	0.006 (−0.465, 0.476)	0.492
2,3-Dihydroxybutanedioic acid	0.85 (0.53, 1.36)	0.286	2.46 (1.19, 5.10)	0.021	0.181 (−0.457, 0.819)	0.321	0.141 (−0.396, 0.677)	0.333
2-Oxovaleric acid	1.36 (0.78, 2.37)	0.179	0.63 (0.29, 1.35)	0.161	0.432 (−0.137, 1.001)	0.106	−0.627 (−1.053, −0.200)	0.008
2-Methylbutyrylglycine	0.64 (0.33, 1.27)	0.142	0.59 (0.23, 1.48)	0.171	0.117 (−0.336, 0.570)	0.336	0.002 (−0.516, 0.520)	0.497
1,3-Diisopropylbenzene	0.78 (0.37, 1.66)	0.297	1.45 (0.83, 2.51)	0.136	0.623 (−0.038, 1.285)	0.061	−0.314 (−0.701, 0.072)	0.091
1,2-Dihydro-1,1,6-trimethylnaphthalene	0.72 (0.37, 1.42)	0.215	1.68 (0.89, 3.15)	0.088	0.490 (0.011, 0.970)	0.046	−0.292 (−0.776, 0.192)	0.161
1-Methylhistamine	1.09 (0.62, 1.90)	0.401	1.30 (0.70, 2.42)	0.239	0.105 (−0.617, 0.827)	0.406	0.039 (−0.475, 0.553)	0.450
[12]-Gingerol	1.20 (0.64, 2.26)	0.318	2.84 (0.99, 8.20)	0.052	−0.272 (−0.862, 0.319)	0.225	−0.146 (−0.71, 0.417)	0.335
(2E)-Decenoyl-ACP	0.42 (0.23, 0.79)	0.012	0.89 (0.40, 1.98)	0.405	−0.036 (−0.718, 0.647)	0.466	0.024 (−0.608, 0.655)	0.475
(10E,12Z)-(9S)-9-Hydroperoxyoctadeca-10,12-dienoic acid	1.25 (0.73, 2.15)	0.247	0.59 (0.27, 1.30)	0.138	0.385 (−0.291, 1.062)	0.174	−0.087 (−0.652, 0.479)	0.401

[a]Sparse group LASSO originally identified 59 individual plasma metabolites with differential posttreatment fold changes between the successful eradication and failed treatment groups. For these metabolites, analysis was performed with logistic regression and generalized estimating equations adjusting for age, gender, and baseline pathology. Logistic regression analyses were conducted to examine the progression of gastric lesions when gastric lesions at the baseline and endpoint were considered. Generalized estimating equation analyses were conducted to examine the progression of gastric lesions according to the trajectory of gastric lesions during follow-up.
